# ATTACHMENT SECURITY AND COPING SKILLS IN INTERACTIONS BETWEEN DAUGHTERS AND THEIR MOTHERS WITH COGNITIVE CHANGE

**DOI:** 10.1093/geroni/igae098.4123

**Published:** 2024-12-31

**Authors:** Dustin Gad, Joan Monin, Jenna Wells

**Affiliations:** Cornell University, Ithaca, New York, United States; Yale School of Public Health, New Haven, Connecticut, United States; Cornell University, Ithaca, New York, United States

## Abstract

The caregiving literature shows that daughters with secure attachment to their mothers with cognitive change report decreased stress and caregiver burden. However, the behavioral patterns that underlie this link remain undiscovered. In the present study, we examined the associations between daughters’ apparent security, an observational attachment code developed by Brooke Feeney and colleagues indicating comfortability and openness, in interactions with their mothers and their tendency to use various coping strategies. Daughter-mother dyads (N = 69) engaged in video-recorded discussions about their concerns regarding their mothers’ memory loss. Apparent security was rated by trained coders. Daughters completed questionnaires about their use of coping styles. Results showed that daughters’ greater apparent security was associated with less use of self-distraction (B = -.470, p =.047), denial (B = -.471, p =.005), and behavioral disengagement (B = -.422, p =.010). Daughter’s greater apparent security was also associated with greater displays of positive affect (r =.487, p <.001), emotional disclosure (r =.352, p =.003), descriptive disclosure (r =.293, p =.015), open discussion (r =.496, p <.001), confidence (r =.262, p =.030), and receptiveness to mothers’ support attempts (r =.476, p <.001). These results suggest that interventions and targeted efforts to make daughters feel more open and accepting with their mothers during discussions about memory loss may increase their ability to cope in the context of their mother’s dementia.

